# Metastatic renal cell carcinoma from a native kidney of a renal transplant patient diagnosed by endoscopic ultrasound-guided fine needle aspiration (EUS-FNA) biopsy

**DOI:** 10.15586/jkcvhl.2015.25

**Published:** 2015-04-20

**Authors:** Yaseen Alastal, Tariq A Hammad, Ehsan Rafiq, Mohamad Nawras, Osama Alaradi, Ali Nawras

**Affiliations:** 1 Department of Internal Medicine, University of Toledo Medical Center; 2 Department of Gastroenterology and Hepatology, University of Toledo Medical Center, 3000 Arlington Avenue, MS 1150, Toledo, OH 43614, USA.

## Abstract

Endoscopic ultrasound-guided fine needle aspiration (EUS-FNA) biopsy sampling of enlarged lymph nodes is increasingly used to diagnose metastatic tumors, especially of the gastrointestinal tract and the lungs. Herein, we describe the diagnosis of metastatic renal cell carcinoma from a native kidney of a 54 year-old male patient, who had a 5-years history of renal transplant, by EUS-FNA of mediastinal and celiac lymph nodes. Histological and immunohistochemical findings confirmed the origin of metastatic tumor. EUS-FNA with proper cytological evaluation can be useful in the diagnosis of metastatic renal cell carcinoma in renal transplant patients.

## Introduction

Endoscopic ultrasound-guided fine needle aspiration (EUS-FNA) biopsy is increasingly used to diagnose metastatic tumors. Lymph node sampling by fine needle aspiration (FNA) offers a chance to evaluate the origin of tumor cells, and subsequently aid the management of such tumors. Herein, we present a case of metastatic renal cell carcinoma (RCC) of the native kidney, in a renal transplant patient, diagnosed by EUS-FNA biopsy of the mediastinal and celiac lymph nodes.

## Case report

**Figure 1.**
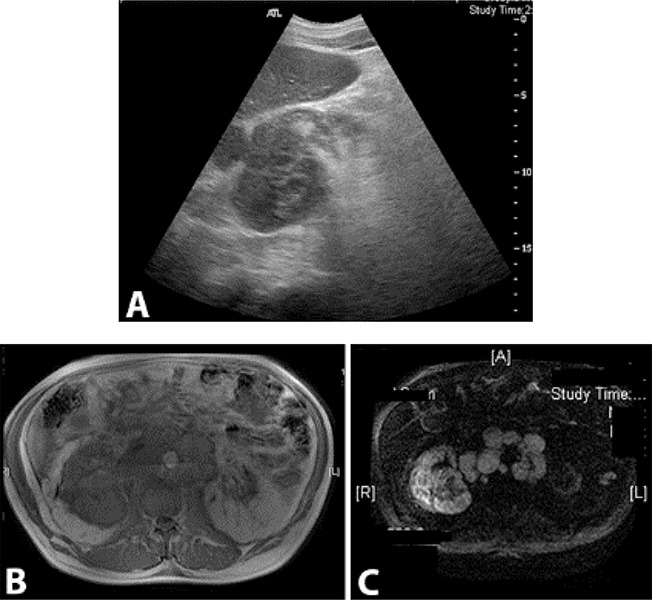
A, Renal ultrasound revealed multiple hypoechoic lesions in the right native kidney. Abdominal MRI showed an enlarged right native kidney with irregular margins (B). Additionally, retroperitoneal adenopathy was observed (C).

**Figure 2.**
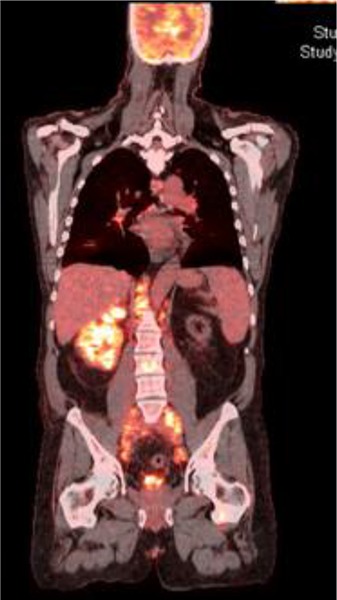
PET scan: Abnormal uptake in T11, L3 and the right acetabulum; significant supraclavicular, mediastinal and retrocrural adenopathy in addition to the extensive retroperitoneal adenopathy; abnormal enlargement of right kidney.

**Figure 3.**
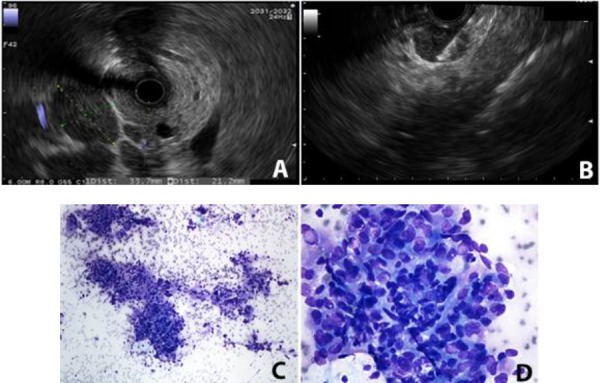
A, EUS showed an enlarged peripancreatic/periduodenal lymph node; B, EUS-FNA of enlarged celiac lymph node; Enlarged and hyperchromatic tumor cells (Diff Quickistain) displaying lack of polarity in low power (C), and high power (D).

Informed Consent was obtained from the patient as per the policy of our institution. A 54 year old male patient with a 5-year history of living-related donor renal transplant, secondary to Goodpasture syndrome, presented with nausea, vomiting and moderate lower abdominal pain for 3 weeks. The patient also reported a 10 lb weight loss in 2 months. He had a history of smoking, 25 packs of cigarettes per year, but quit about 6 years earlier. His father died of renal cancer at the age of 60. At presentation, he was afebrile and normotensive. Abdominal examination revealed a soft non-tender abdomen, with no graft tenderness. Laboratory work-up showed white blood cell counts of 7.4 × 10^9^/L, hemoglobin of 9.5 g/dL, and platelets of 311 x 10^9^/L. His serum creatinine was 3.27 mg/dL (his baseline creatinine was 2mg/dL) while electrolytes were within normal limits. Liver function test, amylase and lipase were all within normal limits. Renal ultrasound revealed multiple hypoechoic lesions in the right native kidney, which were possibly solid in nature (**Figure 1A**). The patient underwent abdominal magnetic resonance imaging (MRI) for further evaluation of these lesions. MRI revealed an enlarged right native kidney with irregular margins. Additionally, retroperitoneal adenopathy was observed below the level of the left renal vein, with largest lymph node measures of approximately 4.7 x 3.8 cm in cross-section below the left kidney (**Figure 1 B, C**). Positron emission tomography (PET) scan revealed significant supraclavicular, mediastinal and retrocrural adenopathy in addition to the extensive retroperitoneal adenopathy. The right native kidney showed abnormal enlargement and increased radiopharmaceutical accumulation which is abnormal in the setting of renal transplant history with atrophic native kidneys. Abnormal uptake was also identified in the T11 vertebral body, L3 vertebral body and in the posterior column of the right acetabulum which could represent bone metastases (**Figure 2**). The patient was scheduled for EUS to evaluate the perigastric/peripancreatic area for possible FNA of lymph nodes. EUS revealed multiple large round and hypoechoic mediastinal lymphadenopathy distributed between the subcarinal and the paraesophageal areas, and at the peripancreatic/periduodenal areas, the largest measured 33.7 mm x 21.2 mm in size (**Figure 3A**). Additionally multiple celiac lymph nodes were identified, the largest measured 20.9 mm x 14.5 mm. EUS-FNA of enlarged celiac lymph node was performed (**Figure 3B**). The histologic features showed enlarged and hyperchromatic tumor cells without polarity (**Figures 3C, D**). Histochemical findings were consistent with metastatic adenocarcinoma of kidney origin (Positive = PAX8 and vimentin; Negative = CK7, CK20, CDX-2, S100, TTF-1, Pan CK, data not shown). The patient was started on chemotherapy and referred for palliative radiotherapy but he decided to discontinue treatment and proceeded with Hospice Care.

## Discussion

RCC is an increasingly incident malignant tumor that accounts for 2–3% of all adult malignancies (1; 2). Smoking is one of the most recognized risk factors for RCC (3). Once patients developed metastatic disease, their prognosis is poor with a median survival of about 13 months (4). About 30% of patients with RCC have metastatic disease at the time of presentation with lungs, lymph nodes, liver, and bone being the common sites (2). Similar to other malignancies, diagnosis mainly relies on tissue sampling of the primary tumor or secondary metastasis. Sometimes, metastatic lesions could be the first presentation of the tumor, and sampling of these secondary lesions could point to the primary origin. With the increasing use of EUS in evaluation and sampling of lymph nodes and gastrointestinal masses, it could also assist in the diagnosis of metastatic RCC. The role of EUS in the diagnosis of metastatic RCC has been discussed in the literature. In most cases, EUS was used to evaluate pancreatic masses, which then were diagnosed as secondary metastasis from RCC. Beáchade et al. used EUS-FNA in 11 patients, who had a history of RCC and a solid mass within the pancreas, and identified 9 of them having pancreatic metastases of RCC (5). Additionally, Waters et al. did a retrospective review of 66 patients who underwent EUS-FNA for tumors that have metastasized to the pancreas and found that the most common site of origin for these metastases was kidney (27 [41%] cases) (6).

Another possible role of EUS in diagnosis of metastatic RCC is sampling of the metastatic lymph nodes. The mediastinum is one of the common sites of metastases from RCC. This makes EUS a proper diagnostic modality in this setting. Fritscher-Ravens et al. used EUS-FNA in 111 patients for evaluation of mediastinal lymph nodes. Seven patients were diagnosed by cytology to have metastatic RCC, and four of them were first diagnosed based on this study (7). This makes EUS-FNA a less invasive technique for evaluation of mediastinal lymph nodes, which then can lead to the diagnosis of metastatic RCC. Additionally, endobronchial ultrasound has been used similarly to diagnose metastatic RCC to the mediastinum (8; 9).

In our case, we used EUS-FNA biopsy to diagnose metastatic RCC. Our patient had radiological finding of multiple enlarged lymph nodes in the mediastinum and abdomen. By using EUS, we were able to access both the mediastinal and celiac lymph nodes and obtain multiple FNAs from both lymph nodes, and we were able to accurately diagnose the site of origin of the metastases. Our case is unique in multiple aspects. First, our patient had a history of renal transplantation, and he developed metastatic RCC of the native kidney. To the best of our knowledge, there are no similar case reports of EUS-FNA of lymph nodes used in the diagnosis of metastatic RCC in renal transplant patients. Studies suggest that kidney cancer is approximately 15-fold more common in renal transplant patients compared to general population, in addition to the increased risk of other malignancies, which could be related to immunosuppressive medications or viral infections (10). This risk, in addition to smoking and family history, may have contributed to the development of RCC in our patient. Second, the extensive lymph node involvement in our patient was more suggestive of lymphoma rather than RCC. EUS-FNA biopsy helped in confirming the diagnosis as metastatic adenocarcinoma of kidney origin.

## Conclusion

EUS-FNA biopsy can be useful in the diagnosis of metastatic RCC in renal transplant patients. It can contribute to diagnosis by sampling mediastinal lymph nodes or pancreatic masses. Proper cytological evaluation can identify the primary source and guide therapy.
